# Optimised Anaesthesia to Reduce Post Operative Cognitive Decline (POCD) in Older Patients Undergoing Elective Surgery, a Randomised Controlled Trial

**DOI:** 10.1371/journal.pone.0037410

**Published:** 2012-06-15

**Authors:** Clive Ballard, Emma Jones, Nathan Gauge, Dag Aarsland, Odd Bjarte Nilsen, Brian K. Saxby, David Lowery, Anne Corbett, Keith Wesnes, Eirini Katsaiti, James Arden, Derek Amaoko, Nicholas Prophet, Balaji Purushothaman, David Green

**Affiliations:** 1 Wolfson Centre for Age-Related Diseases, King’s College London, London, United Kingdom; 2 Department of Neurobiology, Ward and Society, Karolinska Institute, Stockholm, Sweden, Norway; 3 Faculty of Science and Technology, Stavanger University Hospital, Stavanger, Norway; 4 Institute of Ageing and Health, University of Newcastle, Newcastle, United Kingdom; 5 Research Department of Primary Care and Population Health, University College London, London, United Kingdom; 6 Research Directorate, Alzheimer’s Society (UK), London, United Kingdom; 7 Centre for Human Psychopharmacology, Swinburne University, Melbourne, Australia; 8 Department of Anaesthetics, King’s College Hospital, London, United Kingdom; Massachusetts General Hospital, United States of America

## Abstract

**Background:**

The study determined the one year incidence of post operative cognitive decline (POCD) and evaluated the effectiveness of an intra-operative anaesthetic intervention in reducing post-operative cognitive impairment in older adults (over 60 years of age) undergoing elective orthopaedic or abdominal surgery.

**Methods and Trial Design:**

The design was a prospective cohort study with a nested randomised, controlled intervention trial, using intra-operative BiSpectral index and cerebral oxygen saturation monitoring to enable optimisation of anaesthesia depth and cerebral oxygen saturation in older adults undergoing surgery.

**Results:**

In the 52 week prospective cohort study (192 surgical patients and 138 controls), mild (χ^2^ = 17.9 p<0.0001), moderate (χ^2^ = 7.8 p = 0.005) and severe (χ^2^ = 5.1 p = 0.02) POCD were all significantly higher after 52 weeks in the surgical patients than among the age matched controls. In the nested RCT, 81 patients were randomized, 73 contributing to the data analysis (34 intervention, 39 control). In the intervention group mild POCD was significantly reduced at 1, 12 and 52 weeks (Fisher’s Exact Test p = 0.018, χ^2^ = 5.1 p = 0.02 and χ^2^ = 5.9 p = 0.015), and moderate POCD was reduced at 1 and 52 weeks (χ^2^ = 4.4 p = 0·037 and χ^2^ = 5.4 p = 0.02). In addition there was significant improvement in reaction time at all time-points (Vigilance Reaction Time MWU Z =  −2.1 p = 0.03, MWU Z = −2.7 p = 0.004, MWU Z = −3.0 p = 0.005), in MMSE at one and 52 weeks (MWU Z = −2.9 p = 0.003, MWU Z = −3.3 p = 0.001), and in executive function at 12 and 52 weeks (Trail Making MWU Z = −2.4 p = .0.018, MWU Z = −2.4 p = 0.019).

**Conclusion:**

POCD is common and persistent in older adults following surgery. The results of the nested RCT indicate the potential benefits of intra-operative monitoring of anaesthetic depth and cerebral oxygenation as a pragmatic intervention to reduce post-operative cognitive impairment.

**Trial Registration:**

Controlled-Trials.com ISRCTN39503939

## Introduction

Postoperative cognitive decline (POCD) is recognized as a common and impactful outcome of surgical procedures in older adults [1], [2]. POCD is most common after cardiac surgery although up to 40% of people are affected for one week after non-cardiac procedures. Importantly, up to 15% of people continue to be affected after three months [3], [4]. The first major study of long term POCD in people over 60 reported an incidence of 26% one week after surgery and 10% after three months [2]. Whilst the balance of literature supports the persistence of POCD for at least 3 months, not all studies have demonstrated persistent change [5]. A systematic review has highlighted attentional dysfunction as a prominent and sensitive feature of POCD [6]. The clinical significance of POCD, particularly in older indivudals, is emphasised by evidence of the impact it confers on the ability to perform daily activities [7]. Despite the potential long-term implications for patients and health service provision very few studies have assessed POCD for longer than six months post operatively.

The pathophysiology and causative mechanisms for POCD are poorly understood [3]. Risk factors include age, preoperative cognitive function, length of the operation, and postoperative respiratory complications and infection. Proposed interventions to modify these factors include monitoring of cerebral oxygen de-saturation (rSO2) and correcting for duration and depth of anaesthesia [8]–[10]. Significant cerebral oxygen desaturation occurs in up to 30% of patients during major non-cardiac surgery and preliminary evidence indicates that monitoring improves outcome by enabling prompt intervention [8], [11]. Measurement of the bispectral index (BIS, Covidien inc, Co, USA) has been promoted as a variable for depth of anaesthesia. There is high correlation between BIS and clinical criteria of sedation, and a recent review reported that BIS-guided anaesthesia improves outcomes of surgical procedures [12]. It will also be important to identify biomarkers which robustly predict the extent and persistence of POCD. The protein S100B has been identified as a predictor of poor outcome following traumatic brain injury and so is a promising candidate [13], [14].

This 52-week cohort study compared post-operative patients with age-matched controls. This design is important as it addresses the unanswered issue of long term cognitive decline. Within the post-operative group we performed a nested 52-week pragmatic RCT to evaluate combined BIS and cerebral oxygenation (rSO2) monitoring using the Somanetics Invos Cerebral Oximeter (SICO, Covidien inc, Co, USA) to enable optimization of depth of anaesthesia and to identify significant episodes of cerebral oxygen de-saturation indicated by a fall in rSO2. Our hypotheses were that there would continue to be an elevated frequency of impaired cognition after 52 weeks in the surgical patients compared to the age matched community cohort, and that an intervention to enable optimisation of anaesthesia depth and cerebral oxygen saturation in older adults undergoing surgery would improve cognitive outcomes one, 12 and 52 weeks post-operatively.

## Results

### Longitudinal Cohort Study

330 participants were assessed at baseline including 192 surgical patients and 138 controls. The baseline characteristics are reported in Table 1. There was no significant difference in age, baseline MMSE or gender between the two groups.

**Table 1:** Sample Characteristics for the Prospective Cohort Study.

	Patient (n = 192)median (IQR)	Control (n = 138)median (IQR)	Evaluation
**Age**	75 (72 to 81)	76 (72 to 79)	MWU Z = −0.7 p = 0.50
**Pre-Operative MMSE**	28 (27 to 29)	28 (27 to 29)	MWU Z = −1.1 p = 0.25
**Male Gender**	77 (40%)	51 (37%)	χ^2^ = 0.8 p = 0.56

A total of 256 (78%) participants completed the follow-up assessments after 52 weeks. There were no significant differences with respect to age (MWU Z = −0.1, p = 0.91), baseline MMSE (MWU Z = −0.9, p = 0.31) or gender (χ^2^ = 0.8 p = 0.37) between participants who completed the follow-up assessment and those who did not.

### Global Decline

At 52 weeks post-operatively there was a significant difference between patients and controls in all three levels of cognitive decline. Mild POCD (AACD) was identified in 76% of surgical patients compared to 50% of non-surgical controls (χ^2^ = 17.9 p<0.001). Moderate POCD (MMCI) was identified in 34% of surgical patients compared to 18% of non-surgical controls (χ^2^ = 7.8 p = 0.005). Severe POCD was identified in 11.2% of surgical patients compared to just 3.8% of non-surgical controls (χ^2^ = 5.1 p = 0.02).

### 52-week RCT

**Figure 1:**
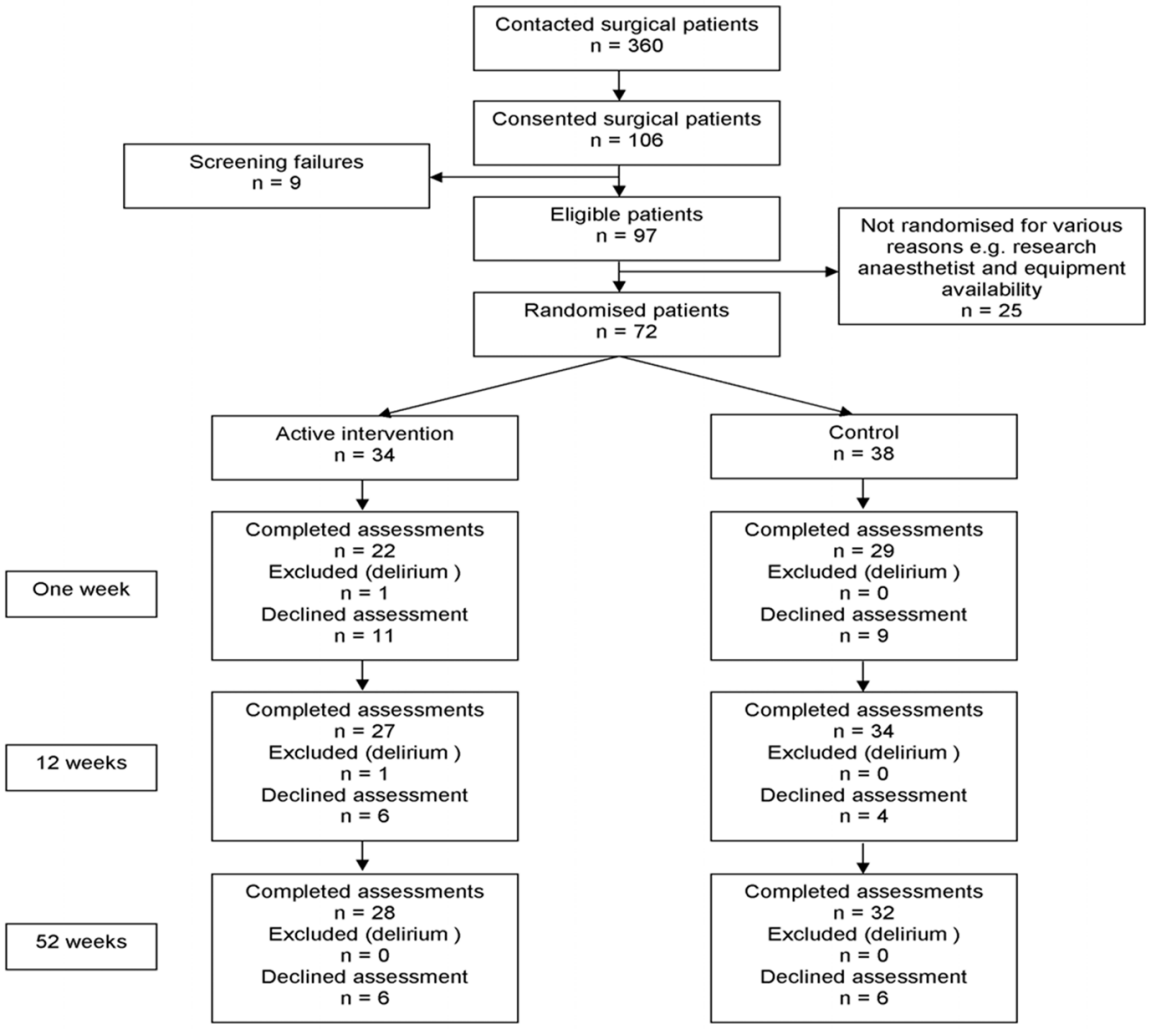
CONSORT chart showing flow of participants through the trial. Assessment of POCD required completion of seven cognitive assessments which were not all completed for all individuals at all timepoints. Presence/absence of POCD was therefore determined as follows throughout the trial. Week 1: Intervention group – 19, Control group – 28; Week 12: Intervention group – 24; Control group – 33; Week 52: Intervention group 27; Control group – 32.

Recruitment for the RCT ran from March 2007 to January 2009, with follow-up of the final participants until January 2010. A total of 72 participants were randomised to the trial. Participant flow is summarised in the CONSORT chart in Figure 1.

There were no significant differences between the intervention and control groups with respect to baseline characteristics (the mean age of participants at the time of surgery, gender, pre-operative MMSE or duration of surgery), the type of surgery received (abdominal, orthopaedic, laparoscopic, open abdominal aortic aneurysm repairs), or key post-operative medications (opioid analgesics; psychotropics, antibiotics) (Table 2).

**Table 2:** Baseline Sample Characteristics for the RCT.

	Intervention mean/median(n = 34) (SD/IQR)	Control mean/median(n = 38) (SD/IQR)	Evaluation
**Age**	75·69 (SD 7·40)	75·16 (SD 6.51)	t −0·3 P = 0·75
**Pre-Operative MMSE**	29 (28 to 29)	29 (28 to 29)	Z −9 P = 0·37
**Duration of Surgery (min)**	127 (108 to 150)	133 (110 to 159)	Z −0·07 P = 0·94
**ASA**	2 (2 to 2.5)	2 (2 to 2)	Z −1·5 P = 0·13
**Gender**			
Male	10 (28%)	12 (32%)	
Female	24 (71%)	26 (68%)	χ^2^ 0·1 df 1 P = 0·75
**Type of surgery**			
Abdominal	6 (18%)	6 (16%)	
Orthopaedic	28 (82%)	32 (84%)	χ^2^ 0·1 df 1 P = 0·74
**Type of Medication**			
% On Opiate analgesics	26 (76%)	27 (71%)	χ^2^ 0·5 df 1 p = 0·50
% On Psychotropic medication	3 (9%)	3 (8%)	Fisher’s Exact test p = 1·00
% On Antibiotics	6 (18%)	3 (8%)	Fisher’s exact test p = 0·28

### Global Cognitive Decline

At one week post-operatively the treatment group showed significant reduction in the number of people developing mild (58% v 89% Fisher’s exact test p = 0·018) and moderate (χ^2^ = 4.4 57% v 26% p = 0·037) POCD. Incidence of severe POCD was numerically lower in the intervention group (5% v 25% Fisher’s exact test p = 0.12).

At 12 weeks the incidence of mild POCD was significantly reduced in the intervention group (54% v 82% χ^2^ = 5.1 p = 0·02). The levels of moderate and severe POCD were not statistically different between the intervention and control groups at this juncture.

At 52 weeks mild and moderate POCD were significantly lower in the intervention group (84% v 56% χ^2^ = 5.9 p = 0·015 and 37.5% v 11.1% χ^2^ = 5.4 p = 0·02). Severe POCD remained numerically lower in the intervention group (12.5% v 3.7% Fisher’s exact test p = 0.36). The results for the comparisons at 1, 12 and 52 weeks are shown in Table 3. It is striking that the rates of severe POCD at 52 weeks in the intervention group appear similar to those in the non-surgical control cohort.

**Table 3:** RCT Outcome Comparisons: Post-Operative Cognitive Decline (POCD).

POCD level at 1 week	Trial Group	No	Yes	Evaluation
**Mild**	controlintervention	10.7% (n = 3)42.1% (n = 8)	89.3% (n = 25)57.9% (n = 11)	**Fisher's exact test p = 0.018**
**Moderate**	controlintervention	42.9% (n = 12)73.7% (n = 14)	57.1% (n = 16)26.3% (n = 5)	**Pearson’s χ^2^ = 4.4 p = 0.037**
**Severe**	controlintervention	75% (n = 21)94.7% (n = 18)	25% (n = 7)5.3% (n = 1)	**Fisher's exact test p = 0.12**
**POCD level at 12 weeks**	**Trial Group**	**No**	**Yes**	**Evaluation**
**Mild**	controlintervention	18.2% (n = 6)45.8% (n = 11)	81.8% (n = 27)54.2% (n = 13)	**χ^2^ = 5.1 p = 0.02**
**Moderate**	controlintervention	72.7% (n = 24)75% (n = 18)	27.3% (n = 9)25% (n = 6)	**χ^2^ = 0.04 p = 0.85**
**Severe**	controlintervention	87.9% (n = 29)91.7% (n = 22)	12.1% (n = 4)8.3% (n = 2)	**χ^2^ = 0.2 p = 0.65**
**POCD level at 52 weeks**	**Trial Group**	**No**	**Yes**	**Evaluation**
**Mild**	controlintervention	15.6% (n = 5)44.4% (n = 12)	84.4% (n = 27)55.6% (n = 15)	**χ^2^ = 5.9 p = 0.015**
**Moderate**	controlintervention	62.5% (n = 20)88.9% (n = 24)	37.5% (n = 12)11.1% (n = 3)	**χ^2^ = 5.4 p = 0.02**
**Severe**	controlintervention	87.5% (n = 28)96.3% (n = 26)	12.5% (n = 4)3.7% (n = 1)	**Fisher's exact test p = 0.36**

POCD at 1 week (n = 19 intervention group, n = 28 control group). POCD at 12 weeks (n = 24 intervention group, n = 33 control group). POCD at 52 weeks (n = 27 intervention group, n = 32 control group).

### Individual Cognitive Domains

The intervention group showed significantly less decline in global cognitive function (MMSE) and reaction time at one week. At 12 weeks the intervention group performed significantly better in reaction time and executive function (Trail Making), but there were no significant differences in MMSE. At 52 weeks, significant benefits were still evident in the intervention arm compared to the non-intervention arm on the trail making test and reaction time. In addition, global cognitive benefits were evident on the MMSE in the intervention group compared to the control group at 52 weeks. The MMSE is an insensitive measure of change and the absence of a difference at 12 weeks was probably due to learning effects. The results are shown fully in Table 4.

**Table 4:** Outcome Comparisons: Cognitive Outcomes (Change from Baseline between groups).

Measures		Intervention	Control	Evaluation
Week 1		Intervention (n = 22)	Control (n = 29)	
**MMSE**	Median (IQR)Mean (SD)	0·0 (−0.25 to 1.00)0.09 (SD 1.60)	−1·0 (−3.0 to 0.0)−2.39 (SD 4.09)	**MWU Z = **−**2·9 P = 0·003** *
**Vigilance** **Reaction** **Time**	Median (IQR)Mean (SD)	−0·01 (−21.88 to 9.65)6.39 (SD 80.90)	27·27 (−21.36 to 70.32)27.95 (SD 54.99)	**MWU Z = **−**2·1 P = 0·03** *
**Week 12**		**Intervention (n = 27)**	**Control (n = 34)**	**Evaluation**
**MMSE**	Median (IQR)Mean (SD)	0·0 (−1.00 to 1.00)−0.04 (SD 1.56)	0·0 (−1·00 to 0.00)−0.79 (SD 2.25)	**MWU Z = **−**1·4 P = 0·16**
**Vigilance** **Reaction** **Time**	Median (IQR)Mean (SD)	−9·76 (−32.54 to 12.60)−11.73 (SD 33.50)	12.61 (−8.58 to 39.27)13.61 (SD 29.69)	**MWU Z = **−**2·6 P = 0·01** *
**Trail Making**	Median (IQR)Mean (SD)	−0·35 (−0.62 to 0.29)−0.23 (SD 0.73)	0·02 (−0.23 to 0.91)0.47 (SD 1.34)	**MWU Z = **−**2·4 P = 0·018** *
**Week 52**		**Intervention (n = 28)**	**Control (n = 32)**	**Evaluation**
**MMSE**	Median (IQR)Mean (SD)	1.00 (0.00 to 1.50)0.69 (SD 1.47)	0.00 (−2.00 to 0.00)−0.94 (SD 2.18)	**MWU Z = **−**2.9 p = 0.004** *
**Vigilance** **Reaction** **Time**	Median (IQR)Mean (SD)	−1.71 (−26.83 to 12.22)−10.80 (SD 36.28)	23.53 (3.29 to 36.22)15.10 (SD 40.73)	**MWU Z = **−**2.8 p = 0.005** *
**Trail Making**	Median (IQR)Mean (SD)	0.31 (−0.49 to 0.55)0.12 (SD 0.68)	−0.32 (−1.07 to 0.24)−0.47 (SD 0.93)	**MWU Z = **−**2.6 p = 0.01** *

Results presented as both median with upper and lower quartiles and mean with Standard Deviation.

MWU – Mann-Whitney U test, SD – Standard Deviation, IQR – Inter Quartile Range.

* Statistically significant (p<0·05).

### BIS

Both the absolute time in minutes spent outside of the target range (Intervention, N = 31, median = 18·0, IQR = 42 v control, N = 38, median = 33, IQR = 94·5, MWU Z = 2.3, P = 0·02) and the ratio of time spent inside and outside the target range (Intervention, Median = 7·2, IQR = 3·47, v Control, Median = 3·38, IQR = 1·84, Z = 1·99, P = 0·047) were significantly improved in the intervention group. Time spent outside of the target BIS range was also significantly correlated with MMSE one week post surgery (MMSE, R = −0·29, P = 0·04) and with trail making at 12 weeks (R = −0.35 P = 0.013) and at 52 weeks (R = −0.295 P = 0.049).

### rSO2 Differences

In the intervention group there were five incidences of significant (>15%) de-saturation, of which three were successfully treated by the intervention algorithm. There was only one de-saturation below 50%. In the control group there were seven significant de-saturations of which six included an rSO2 below 50% (3·3% v 17·1%, Fisher’s exact test P = 0·11). Of the patients who showed an rSO2 below 50% and who were assessed at one week, all but one met the criteria for severe POCD (9·8% rSO2>50% v 75% rSO2<50%, Fisher’s exact test P = 0·009).

At 12 weeks there was no significant difference between patients who recorded an rSO2<50% with respect to development of POCD. At 52 weeks patients who had experienced an rSO2<50% were significantly more likely to have severe POCD (37.5% v 4.3% Fisher’s Exact test p = 0.018).

### S100B

S100B levels were correlated with both duration of surgery (Spearman’s r = 0·27, P = 0·048) and the number of minutes during which the BIS index was outside the target range (Spearman’s r = 0·42, P = 0·002).

## Discussion

The cohort study confirms that there is a significant increased frequency of cognitive impairment in people over the age of 60 undergoing major non-cardiac surgery, and highlights that a greater level of cognitive impairment is still evident after 52 weeks in comparison to age-matched controls. These results emphasise the clinical importance of POCD and its long term impact on cognition in this patient group.

The ISPOCD study group identified severe POCD in 10% of patients at 12 weeks post operatively and 2.8% of controls. In our prospective cohort study we identified severe POCD in 11.2% of surgical patients compared to just 3.8% of non-surgical controls at 52 weeks post operatively, whilst the RCT demonstrated severe POCD in 12.5% of the non intervention surgical patients at the 12 week analysis. Previous literature on Age Associated Cognitive Decline in adults over 60 has demonstrated the development of AACD in 54% of normal subjects over 1 year – which is consistent with the level of mild POCD identified in our control group [27].

The prospective cohort study is the first study to identify significantly greater levels of global cognitive decline in surgical patients one year postoperatively. This finding supports the literature demonstrating a persistent impact of POCD with respect to patient mortality and welfare over longer term follow-up [28].

The use of a computerised test battery allows for the identification of subtle cognitive deficits with excellent test/retest validity whilst minimising the learning effects found in POCD and may have contribute to the identification of POCD at 52 weeks postoperatively.

The pilot nested RCT reports the first successful intervention to reduce cognitive impairment after major non-cardiac surgery in older people. The RCT indicated that a pragmatic intervention to optimize anaesthetic depth and maintenance of cerebral oxygenation with BIS and SICO monitoring conferred a significant reduction in mild and moderate POCD, global cognitive impairment and attentional impairments at one week post-surgery. The intervention also conferred sustained benefits in reducing mild POCD and improving attention, executive performance and global cognition up to 52 weeks following surgery. The statistical power was limited, particularly to detect differences in severe POCD, but the frequencies were lower in the intervention group and the overall pattern of results indicated clear benefit in the intervention group. It is notable that in this pilot study, the intervention appeared to reverse the risk of cognitive impairment at 52 weeks to a level comparable to the non surgical control population.

These findings are consistent with the existing literature although few studies have specifically investigated the effect of targeting BIS levels to reduce the incidence of POCD. This lack of research is surprising since a growing body of evidence from cell and aged animal models (including primates) suggests that general anaesthetics can be neurotoxic to the developing and aging brain. Recent reviews have concluded that despite this evidence it would be premature to change clinical practice since the effect has not been adequately studied in humans [29], [30] and due to robust conflicting evidence that anaesthetics are well known as neuroprotectants and may mitigate against the CNS inflammatory response to surgical trauma [31], [32]. This conflict is resolved by the hypothesis that an optimal range of “depth of anaesthesia” could be identified to maximise neuroprotection and limit neurotoxicity. Elsewhere, evidence also supports the usefulness of BIS monitoring in identifying patients with a good chance of recovery after ischemic-hypoxic brain injury requiring emergency surgery [28], [33]. In the current study we focussed on potentially modifiable intraoperative interventions but it is of course important to interpret this in the context of broader risks including factors leading to altered individual susceptibility [34].

In the current study, patients receiving the monitoring intervention were within the optimal BIS target range (40–60±5) for a significantly higher proportion of the intra-operative period and there was a significant relationship between the proportion of the intra-operative period spent in this optimal window and both cognitive outcome and S100B, a marker of brain injury. It should be acknowledged that the strength of the correlations are modest. However this provides evidence that the BIS monitoring was a key component of the intervention.

A further important question is whether SICO monitoring also contributed to the benefits conferred by the intervention in this study. There were numerical differences in the number of patients experiencing significant cerebral oxygen de-saturation in the two groups, the majority occurring in the control group. As this difference did not achieve statistical significance caution must be taken in interpreting this outcome. However, it upholds the rationale that SICO monitoring may make an important contribution in a modest sub-group of patients. For example, in the control group six patients suffered cerebral oxygen desaturations below 50% and this was associated with an increased risk of POCD at one week. In comparison, only one patient experienced this level of cerebral oxygen desaturation in the intervention group. This is consistent with findings from a previous study [7], [8] which reported that patients in a control group with intraoperative cerebral desaturation experienced a longer time to post-anaesthesia care unit (PACU) discharge and longer hospital stay compared with patients of the treatment group. The study concluded that using rSO2 monitoring to manage anaesthesia in elderly patients undergoing major abdominal surgery reduces the potential exposure of the brain to hypoxia, and postulated that his might be associated with decreased effects on cognitive function and shorter PACU and hospital stay [7], [8]. Although this requires further investigation, it is therefore possible that the SICO monitoring may have contributed to the impact of the intervention in a sub-group of participants in the current study.

This study used robust definitions of mild, moderate and severe POCD, based upon Z scores across several cognitive domains, using a threshold of 1–2 SD, derived from a pre-trial cohort study. This approach combines the rigour of previous POCD studies, but also incorporates measures of mild and moderate POCD (equivalent to AACD and MMCI, respectively) to enable the magnitude of cognitive dysfunction and the potential risk of subsequent dementia to be interpreted in the context of other studies evaluating cognitive dysfunction in older people.

The results of the cohort study and RCT highlight persistent impairments of attentional and executive function after major-non cardiac surgery. Interestingly, other studies examining POCD have indicated that these aspects of cognitive dysfunction are more closely linked to impairments of Instrumental Activities of Daily Living (IADL) than impairments in other cognitive domains [19], [29], emphasising the potential importance of this pattern of cognitive deficits. Key questions pertain to whether this pattern of persistent post-operative cognitive deficits indicates a significant risk for progressive cognitive decline and dementia over longer term follow-up.

Although the RCT was based on a stipulated power calculation, the sample is modest in size. In addition as this was an acute post-surgical study there were a substantial minority of participants who were not able to complete the one week assessments and this may have introduced a bias. In addition, multiple statistical evaluations were undertaken. The data do therefore need to be interpreted with some caution. The outcome is however extremely encouraging and indicates the exciting possibility that a simple and pragmatic monitoring intervention can significantly reduce post-operative cognitive impairment. A larger replication study is urgently indicated and further work to evaluate the impact on resource utilization will be valuable to determine the potential cost-effectiveness.

The study highlights significant and persistent cognitive deficits in older people after major-non cardiac surgery. A preliminary RCT suggests that a pragmatic intervention focussing upon BIS and rSO2 can significantly reduce cognitive impairment at one, 12 and 52 weeks post-operatively.

## Methods

The protocol for this trial and supporting CONSORT checklist are available as supporting information; see Checklist S1 and Protocol S1.

### Study Design and Objectives

The study was conducted in two parts. A prospective longitudinal cohort study was conducted in order to (1) quantify cognitive decline over a 52 week period comparing a post operative group with an age, gender and intelligence matched sample. (2) to determine the sensitivity of the cognitive test battery, including the computerised CDR assessment for the identification of POCD.

Within the prospective cohort study a nested 52 week parallel group, double blind, 1∶1 randomized controlled trial, comparing active anaesthetic monitoring with a control condition was conducted. To determine whether an intervention to enable optimisation of anaesthesia depth and cerebral oxygen saturation (rSO2) in older adults undergoing surgery conferred benefit with respect to cognitive outcomes at one, 12 and 52 weeks post-operatively in comparison to usual treatment. The primary outcome measures were development of mild, moderate and severe POCD and one, 12 and 52 weeks. Changes in specific cognitive evaluations were also compared between groups. An additional analysis examined concentrations of S100B at baseline and 24 hours post-surgery to determine whether it is a reliable predictor of outcome in older people.

### Recruitment and Inclusion Criteria

Patients from two UK regional teaching hospitals were matched to control participants from the local regions based on age, gender and baseline score in the Mini Mental State Exam (MMSE 15). In Newcastle control participants were recruited from randomly selected people over the age of 60 from GP lists. In London controls were identified from spouses of participants. The control population was stratified into 5 year age bands from which individuals were randomly selected to match the number of patients in the postoperative cohort within each 5 year age band. The health of the controls is likely to reflect that of the general population for this age.

Patients were eligible for inclusion if they were scheduled to undertake elective major abdominal or orthopaedic surgery under general anaesthesia at either centre and were over 60 years of age, classified as American Society of Anesthesiologists (ASA) as class three or less and had an MMSE score equal to or greater than 23. Adequate English was a pre-requisite to enable consent and to undertake study assessments.

### Exclusion Criteria

Participants were excluded if they were unable to complete the outcome measures, they had a diagnosis of Alzheimer’s disease or other dementia, if they were undergoing surgical procedures under regional anaesthesia or if they had delirium at 1 week post surgery.

### Randomisation and Blinding

The nested RCT trial was double-blinded; patients and researchers collecting outcome data were blind to treatment allocation. Only the anaesthetist delivering the intervention was aware of the treatment condition. Participants were randomized according to randomisation lists, generated by the study statistician in the statistical program package R, which were stratified by age group (65 to 70, 70 to 75 and over 75). Sealed envelopes containing the randomization codes were delivered to operating theatres, and an envelope selected randomly by the anaesthetist. The randomisation envelope was opened only after the participant’s eligibility and willingness to participate were re-confirmed prior to surgery.

**Figure 2:**
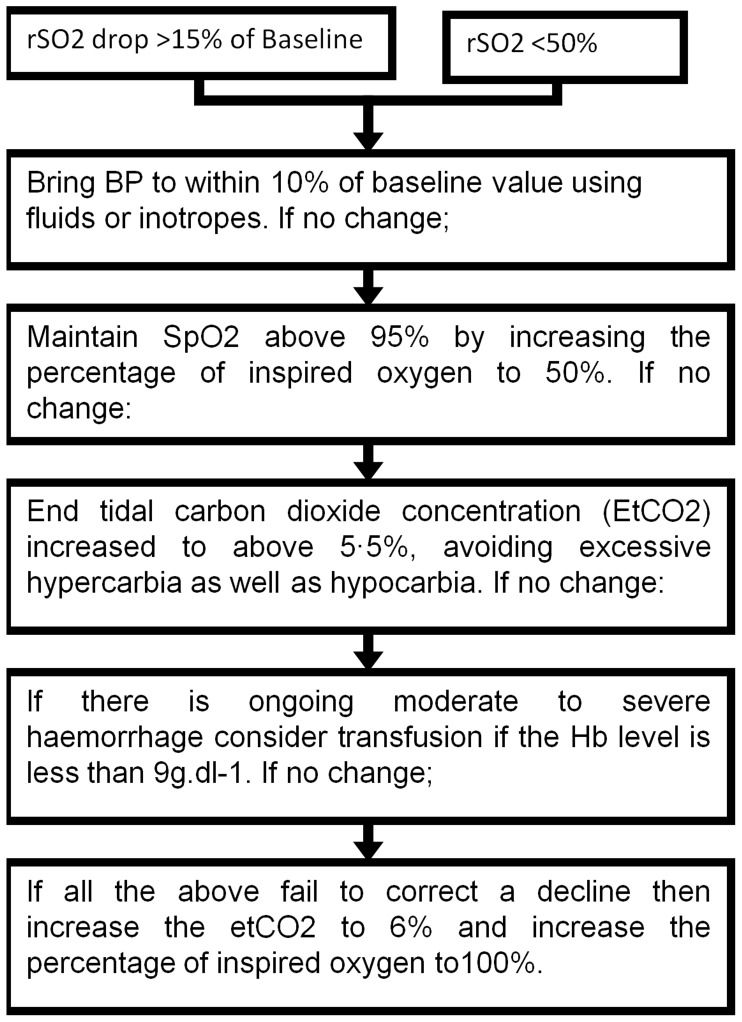
Protocol for anaesthetic intervention.

### Assessments and Outcomes

Patients were assessed using a combination of the MMSE [15] and the three automated tests of attention from the Cognitive Drug Research (CDR) system, simple reaction time, choice reaction time and digit vigilance [16]–[18]. Assessments were carried out at baseline (preoperatively for surgical patients) and at 12 months following the baseline assessment for controls. In the RCT arm assessments were also conducted at 1 and 12 weeks post baseline assessment. Change over time was measured in relation to the baseline assessment. The CDR battery was selected as a sensitive and well validated evaluation of attentional performance [16], which has previously been shown to be sensitive to cognitive impairments in post-operative patients [17], [18]. Computerised testing has the added advantage of excellent test/retest validity and the use of this attentional battery minimises learning effects.

Post operative cognitive decline was classified at three levels - mild, moderate and severe, building upon previous work focussing on POCD [19] These levels correspond to measures commonly used to describe cognitive decline in older people, specifically, age-associated cognitive decline (AACD), multi-domain mild cognitive impairment (MMCI) and post-operative cognitive dysfunction (POCD) [20]–[23]. The scores were calculated using the ISPOCD Z score method described previously [2]. The change for individual participants from baseline was calculated and the control group was used to remove any learning effect and to provide the standard deviations for the Z score calculations. The following seven cognitive measures were used to analyse the level of cognitive decline: MMSE, simple reaction time, digit vigilance accuracy, digit vigilance reaction time, choice reaction time accuracy, choice reaction time and cognitive reaction time. Mild POCD (equivalent to AACD) was defined as a decline in performance in at least one of the seven cognitive domains by greater than one standard deviation (SD). Moderate POCD (equivalent to MMCI) required an additional decline of at least 1.5 SD in an additional domain, and severe POCD was defined as a decline of greater than 1.96 SD in at least two domains. The Confusion Assessment Method (CAM) was used to identify participants with clinically significant delirium.

In the RCT arm BIS values were compared between groups according to absolute time (minutes) spent outside of the optimal range 40–60 (±5). Changes in SICO rSO2 values were calculated. The percentage drop from baseline rSO2 was calculated for each 30 second period.

Plasma levels of the S100B protein were analysed as a biomarker of brain injury [24]. Blood samples were taken from participants at baseline and one day post-surgery. Blood was collected into K2-EDTA vacutainers and centrifuged at 2,000 g for ten minutes. Plasma was collected and frozen until analysis. S100B concentrations were measured using a commercially available ELISA (Alpco Diagnostics, Salem, NH, USA) carried out to the manufacturer’s instructions.

### Statistical Analysis

The RCT was powered *a priori* as a pilot study. A cohort of 73 patients gives 80% power to the 5% level of significance to detect a between group difference of 0·33 SD. This threshold was chosen as the usual one for a meaningful clinical difference i.e. between 0.3 and 0.4 difference in SD [25]. The primary efficacy population was predefined as all patients who were randomised to receive the intra-operative anaesthetic intervention or treatment as usual and completed at least one follow-up assessment.

Analysis was conducted using SPSS software, version 17.0 (SPSS, Chicago, IL, USA). Significance was determined as two-sided p values <0·05. No correction was made for multiple comparisons. Comparison of the baseline MMSE, age and gender of the control and surgical groups was made using the χ^2^ test, Student’s *t* test and Mann–Whitney *U* test as appropriate. The Kolmogorov-Smirnov test was used to establish deviation from a normal distribution. The primary outcome measure was predetermined as the difference in the prevalence of mild, moderate and severe cognitive decline between surgical patients and controls assessed using the χ^2^ test. Continuous analysis was also conducted on the individual cognitive domains using Student’s *t* test, the Mann–Whitney *U* test as appropriate. Demographic factors and clinical characteristics were summarised as counts (percentages) for categorical variables, mean (standard deviation [SD]) for normally distributed continuous variables, or median (interquartile or entire range) for other continuous variables. Correlation Analysis was conducted to examine the relationship between the intra-operative monitoring data and the cognitive outcome variables. Pearson’s or Spearman’s rank correlation analysis was used depending on the distribution of the data.

### Intervention

All patients received intravenous induction of anaesthesia with propofol and maintenance with isoflurane. Depth of anaesthesia and rSO2 were monitored in all participants using BIS and SICO respectively [26]. Monitoring was performed throughout the procedure until extubation. In the intervention group this data was made available to the anaesthetist to enable rigorous optimisation of the depth of anaesthesia and to identify and facilitate additional interventions to rectify changes in cerebral oxygen desaturation (Figure 2). In the control group the BIS and SICO data were collected, but the anaesthetist was blinded to the monitoring data.

The target optimum BIS range was 40–60 (±5).

### Ethical Approval, Consent and Governance

Approvals for ethics and research and development were granted by the Newcastle and North Tyneside research ethics committee and King’s College Hospital NHS Foundation trust under the auspices of the Central Organization of Research Ethics Committees (COREC, now IRAS). Participants were consented using the COREC approved process. King’s College London acted as the sponsor, advised by an independent Data Monitoring Committee (DMC) which was charged with overseeing patient safety. The ISRCTN number for the trial is ISRCTN39503939.

## Supporting Information

Checklist S1 CONSORT checklist.
(DOC)

Protocol S1 Trial protocol.
(DOC)
