# Correction: Optimised Anaesthesia to Reduce Post Operative Cognitive Decline (POCD) in Older Patients Undergoing Elective Surgery, a Randomised Controlled Trial

**DOI:** 10.1371/annotation/1cc38e55-23e8-44a5-ac2b-43c7b2a880f9

**Published:** 2012-09-19

**Authors:** Clive Ballard, Emma Jones, Nathan Gauge, Dag Aarsland, Odd Bjarte Nilsen, Brian K. Saxby, David Lowery, Anne Corbett, Keith Wesnes, Eirini Katsaiti, James Arden, Derek Amoako, Nicholas Prophet, Balaji Purushothaman, David Green

The twelfth author's name was spelled incorrectly. The correct name is: Derek Amoako.

