# Correction: Optimised Anaesthesia to Reduce Post Operative Cognitive Decline (POCD) in Older Patients Undergoing Elective Surgery, a Randomised Controlled Trial

**DOI:** 10.1371/annotation/c0569644-bea1-4c38-af9a-75d1168e3142

**Published:** 2013-09-10

**Authors:** Clive Ballard, Emma Jones, Nathan Gauge, Dag Aarsland, Odd Bjarte Nilsen, Brian K. Saxby, David Lowery, Anne Corbett, Keith Wesnes, Eirini Katsaiti, James Arden, Derek Amaoko, Nicholas Prophet, Balaji Purushothaman, David Green

Multiple funding organizations were incorrectly omitted from the Funding Statement. The Funding Statement should read: "The authors would also like to acknowledge the National Institute for Health Research (NIHR) Mental Health Biomedical Research Centre and Dementia Unit at South London and Maudsley NHS Foundation Trust and [Institute of Psychiatry] King's College London. This article presents independent research funded by the National Institute for Health Research (NIHR). The views expressed are those of the author(s) and not necessarily those of the NHS, the NIHR or the Department of Health." 

