# Supplementary material for: Optimised Anaesthesia to Reduce Post Operative Cognitive Decline (POCD) in Older Patients Undergoing Elective Surgery, a Randomised Controlled Trial
Source: PLoS One. 2012 Jun 15;7(6):e37410. doi: 10.1371/journal.pone.0037410 (PMC3376123; doi:10.1371/journal.pone.0037410)
Supplement: Protocol S1 — Trial protocol. (DOC) [file pone.0037410.s002.doc]

**Postoperative Cognitive Function Research Project [09/07/06 Version 1]**

**Based at King’s College Hospital in collaboration with the Wolfson Centre for Age Related Diseases, KCL, Guy’s Campus**

**A PROSPECTIVE, RANDOMIZED, CONTROLLED EVALUATION OF THE EFFECTS OF OPTIMISATION OF CEREBRAL OXYGENATION AND DEPTH OF ANAESTHESIA ON POSTOPERATIVE COGNITIVE DYSFUNCTION IN ELDERLY PATIENTS UNDERGOING MAJOR ORTHOPAEDIC AND ABDOMINAL SURGERY**

**Synopsis of project: purpose, study design**

Over the last decade, key studies have highlighted the high frequency and persistence of Postoperative Cognitive Decline (POCD), with preliminary studies indicating a particularly high incidence in the oldest age groups. POCD refers to the adverse neurological and neurocognitive changes that many individuals experience after surgery. Impairments particularly seen are short-term or long-term memory and attentional dysfunctions, disturbances in executive functioning and receptive language impairments. POCD is a major, potentially preventable and often unrecognized cause of cognitive decline, but many of the key questions regarding the full significance of the syndrome remain unanswered. For example, the risks of Mild Cognitive Impairment (MCI) and dementia have not been established. An improved understanding of POCD is essential to enable clear communication of the likely risks and prognosis to older surgical patients. investigate the role of potential intra-operative interventions Even more importantly, elucidating the key contributing factors and the underlying mechanisms and developing effective strategies for prevention and treatment. The current study will determine the clinical outcome of POCD, evaluate candidate clinical and biological markers and and genetic mechanisms.

**Details of the proposed investigation**

1. **Title**: **A PROSPECTIVE, RANDOMIZED, CONTROLLED EVALUATION OF THE EFFECTS OF OPTIMISATION OF CEREBRAL OXYGENATION AND DEPTH OF ANAESTHESIA ON POSTOPERATIVE COGNITIVE DYSFUNCTION IN ELDERLY PATIENTS UNDERGOING MAJOR ORTHOPAEDIC AND ABDOMINAL SURGERY**

2. **Research objectives**: The current proposal will determine the frequency and importance of Post-Operative Cognitive Decline (POCD) in older people (over the age of 65), determine the subsequent incidence of Mild Cognitive Impairment and dementia and evaluate the role of key peri-operative intervention strategies and biological risk factors which will enable the development and implementation of preventative therapeutic strategies.

3. **Background**: Clinical experience has always suggested that older people often develop impairments of cognitive function after major surgical interventions. It is however only over the last decade that systematic studies have investigated the problem, coining the term Postoperative cognitive decline (POCD). This body of work has highlighted the frequency and importance of POCD, which has become increasingly recognized as a common and potentially handicapping outcome after a broad range of major surgical procedures. Although there is some variability between reports, probably reflecting the individual study definitions of POCD and the differing age range of participants, the mean reported incidence rates are 25% (range 7 - 55%) at 1 week post-operatively and 10% (range 7-15%) at 3 months post-operatively for non-cardiac procedures. These findings clearly indicate that POCD is a frequent and often persistent syndrome at all ages.

The first major multi-centre international study of long term POCD in people aged 60 and over (The International Study of Postoperative Cognitive Dysfunction, ISPOCD1 study^1^) reported an incidence of POCD of 25% 1 week after surgery and 10% after 3 months, despite excluding people with any pre-existing level of cognitive impairment from the trial. Importantly, the degree of POCD identified was of sufficient magnitude to interfere with day-to-day activities, emphasising the clinical significance of the problem. Furthermore, patients aged 70 and over had twice the incidence of POCD at 3 months (14%) than those aged 60 to 69 (7%). There are very few studies in patients aged 70 or older, who are probably the highest risk group; and only a handful of studies with periods of follow-up longer than 3 months. In a 12 month study undertaken by our group (appendix 1), including 100 participants over the age of 70 undergoing elective orthopaedic surgery, only 45% regained their full level of pre-operative cognitive performance over the subsequent 12 months, even when excluding individuals who developed delirium. Furthermore, people >80 were four times less likely to regain their full level of cognitive functioning than individuals between the ages of 70 and 80, suggesting that the risk of POCD continues to escalate with advancing age even over the age of 70. In addition, specific persistent deficits of attention were evident, particularly the speed of performance on attentional tasks. It is clear from these studies that some level of cognitive impairment is extremely frequent after major surgical procedures, especially in the oldest age groups. Surprisingly, despite the high incidence rates, awareness is low amongst the medical community and amongst surgical patients; and patients are rarely informed of the risk of POCD preoperatively as part of the consent procedure^1^.

POCD is a major and potentially preventable cause of persistent cognitive decline, which often goes unrecognized. Studies so far have only “scratched the surface”, with many key questions regarding the full significance of the syndrome remaining unanswered. For example the proportion of people with persistent cognitive impairment has not been determined, it is unclear whether the magnitude of impairment is of sufficient severity to meet criteria for clinically significant syndromes such as Mild Cognitive Impairment (MCI) and the risk of incident dementia in the aftermath of surgery and over longer term follow-up has not been clarified. In addition, it is important to determine the extent to which POCD occurs independently of delirium, and whether the causes are different in people with or without delirium. Furthering our understanding of the syndrome is essential to enable clear communication of the likely risks and prognosis. Even more importantly elucidating the key contributing factors and the underlying mechanisms is essential so that effective prevention strategies can be devised and implemented.

**4. Causes and potential clinical determinants of risk for POCD**

Peri-operative factors: Several recent editorials have further reiterated the magnitude of the problem but have been unable to offer any insights into the mechanisms or underlying causes^2,3^. For example, in the ISPOCD1 study trial neither hypoxaemia nor hypotension were identified as significant risk factors, even with continuing measurements for up to 3 days post surgery^1^. It is however likely that the monitoring techniques employed in the ISPOCD trial lacked sufficient sensitivity to identify some of the important peri-operative changes and recent technological developments offer an exciting opportunity to improve the quality of peri-operative monitoring, enabling more precise measurement of regional cerebral oxygen saturation (rSO2), depth of anaesthesia and circulatory homeostasis.

a) Cerebral oximetry using the Somanetics Invos Cerebral oximeter (SICO)

The Somanetics Invos Cerebral oximeter (SICO, Somanetics coorporation, Troy, Mich. USA)) is a commercially available device that measures regional cerebral oxygenation (rSO2) and quantifies changes in the balance between cerebral oxygen delivery and consumption. Evidence is also now accumulating in patients undergoing carotid endarterectomy, cardiopulmonary bypass, liver transplantation, neurosurgery and major abdominal surgery that a fall in rSO2 is associated with major cerebral dysfunction and/or postoperative cognitive dysfunction (POCD). Strategies to correct these falls in rSO2 may lead to improved cerebral function and a reduced incidence of POCD.

Although it is not absolutely clear what level of fall is significant, it is known from patients undergoing awake carotid endarterectomy surgery that a fall in rSO2 of greater than 20 per cent following carotid clamping is associated in most patients with marked deterioration in cerebral function. This usually necessitates the insertion of a shunt for the operation to proceed ^4^.

Falls in rSO2 during cardiac surgery are significantly correlated with POCD and correction of these falls is associated with a reduced incidence of POCD^5-7^. The significant falls in rSO2 during cardiac surgery are almost always seen during the hypotension and haemodilution on initiation and continuation of cardiopulmonary bypass which clearly represents a major change in circulatory physiology^8^. However, major blood loss and fluid shifts doing other types of surgery may also cause similar falls in rSO2 sufficient to cause POCD, especially in the elderly.

A significant fall in rSO2 during the anhepatic phase of liver transplantation has been associated with raised postoperative levels of neuron-specific enolase (NSE) and S-100, which are specific variables that indicate cerebral disturbances due to hypoxia/ischemia^9^.

Changes in rSO2 have been shown to be a useful guide to vasodilator management of cerebral vasospasm following aneurysmal subarachnoid haemorrhage^10^.

To evaluate whether monitoring and normalization of cerebral oxygen saturation (rSO2) minimizes intraoperative cerebral desaturation, 122 elderly patients undergoing major abdominal surgery with general anaesthesia were studied^11^. Patients were randomly allocated to an intervention group (the monitor was visible and rSO2 was maintained at > 75% of pre-induction values; *n* = 56) or a control group (the monitor was blinded and anaesthesia was managed routinely; *n* = 66). Cerebral desaturation (rSO2 reduction > 75% of baseline) was observed in 11 patients of the treatment group (20%) and 15 patients of the control group (23%) (*P* = 0.82). To assess whether cerebral desaturation was associated with POCD, the mini-mental state examination (MMSE) was assessed. Although there was no overall statistical difference in outcome (as assessed by reduction in MMSE between the two groups), those patients who suffered a cerebral desaturation had a significantly lower MMSE score at 7 days. A decline in cognitive function (defined as a reduction in MMSE => 2 points from baseline) during the first week after surgery was observed in 20 patients of the treatment group (35%) and 30 patients of the control group (54%) (*P* _ 0.137); however, when considering only those patients who actually had at least one episode of intraoperative desaturation in both groups, a decline in cognitive function was observed in 10 patients of the control group only (66%) (*P* < 0.001). There was also a relationship between the extent of cerebral hypoxaemia and increased length of stay.

However, this study can be criticised on several grounds. Firstly, the MMSE is not a very good discriminator of POCD. Secondly, changes in haemoglobin as a result of blood loss were not recorded. Thirdly, they resorted to the use of propofol to lower cerebral metabolic rate for oxygen (CMRO2) to improve rSO2 rather than using methods such as blood transfusion and optimization of fluid status to improve cerebral oxygen delivery. Nevertheless, they showed that in elderly patients undergoing major surgery there is a risk that falls in cerebral oxygenation may take place which could result in POCD. Overall, though, the incidence of POCD in the treatment arm was still high at 35%, higher than the overall figure of 26% in the ISPOCD1 trial.

b) Depth of anaesthesia and potential toxicity of anaesthetic agents

Another key peri-operative risk factor for POCD pertains to the type and dose of the anaesthetic agents employed. Recent work has demonstrated that most anaesthetic agents may be toxic, in both the developing and the aged rat brain^12,13^. It is hypothesised that the quantity (duration times concentration) of anaesthetic agents used, especially during major surgery for older people, may be an important determinant of POCD^14^. Using the Bispectral Index Monitor [BIS monitor] (Aspect Inc, USA), recent work has demonstrated that cumulative deep hypnotic time (defined as the total amount in time in hours that the BIS level was below 45) is an independent risk factor for poor long term outcome after major non-cardiac surgery^15^. There may also be important differences between agents used to maintain anaesthesia. For example, opioids are non-toxic so the profound MAC sparing ability of remifentanil ^16^may help minimize toxicity of both inhalation and intravenous anaesthetics.

**5. Retrospective analysis of data to assess the role of the above interventions**

Our group has recently completed a retrospective analysis of changes in rSO2 in 46 older patients (mean age 65) undergoing abdominal surgery using the SICO. Twenty three (50%) of the patients had a drop in rSO2 of at least 15% and this was associated with ongoing major blood loss and low Hb and HCT. These changes persisted, despite optimization of anaesthetic depth (using the BIS monitor), arterial oxygenation (using pulse oximetry) and circulatory status using CVP and BP. In all cases, the fall in rSO2 could only be reversed by blood transfusion and with conventional monitoring would have gone unnoticed. As mentioned above, previous studies in awake patients undergoing carotid endarterectomy under local anaesthesia have demonstrated that a fall in rSO2 of this magnitude is often associated with substantial cerebral dysfunction^4^. An initial hypothesis is that these previously undetected falls in rSO2 represent a major and potentially preventable risk factor for POCD. However, other workers have demonstrated that falls in rSO2 as a result of haemodilution are not necessarily mirrored by falls in jugular venous oxygen saturation (SjO2), a measure of **global** cerebral hypoxaemia^17^. This could either be due to a greater sensitivity of the SICO versus SjO2 to reflect localised cerebral hypoxaemia^8^.

**6. Mechanisms and biological markers**

Several recent studies have highlighted potentially important biological markers of POCD, including serum levels of the S-100 protein and Neuron Specific Enolase (NSE) ^18^as well as plasma concentrations of NO products^19^. S100b is a calcium-binding protein, localised predominantly in astroglial cells. Following cellular injury S100b leaks into the extracellular space ^20^and provides an established marker of cerebral tissue damage and cerebral ischaemia ^21,22^. At nanomolar concentrations S100b promotes neurite outgrowth, whereas at micromolar concentrations pro-inflammatory cytokines are expressed and apoptosis is induced^23^. NSE is released during neurological injury and has already been studied as a marker of neonatal^24^ and postoperative neurological injury (Wimmer-Greinecker et al. 1998). Ischaemia results in glutamate release from injured cells with the consequent activation of glutamate receptors. This leads to calcium influx and the activation of nitric oxide synthase (NOS). Neuronal damage may occur directly due to NO or to the peroxynitirite ion, produced by the combination of NO and superoxide^25^. NO levels may be decreased in response to the magnitude and duration of surgical stress^26^, possibly mediated by the inflammatory response^27^. Neurons which express nitric oxide synthase (NOS) appear to be less susceptible to injury^28^. The latter finding is of particular interest, as a genetic polymorphism of ecNOS predisposes to cardiovascular disease and is a significant risk factor for subsequent dementia in people with cerebrovascular disease (appendix 3).

Other candidate genetic factors which may increase vulnerability to POCD include polymorphisms that increase risk of dementia (eg APOE4) and polymorphisms associated with delirium (eg NMDAR1 G2108A polymorphism, an insertion/deletion polymorphism in the promoter region NQO2 and a variable number of tandem repeats (VNTR) polymorphism in the 3’ non-coding region of the dopamine transporter DAT in patients with a history of delirium tremens during alcohol withdrawal). It is also possible that other genetic factors may be protective, for example a common candidate polymorphism that is associated with improved attentional performance may reduce the risk of POCD (BuChE K variant). We therefore hypothesise that particular individuals could be at greater risk of POCD based upon genetic vulnerability; and that there may be an interaction of peri-operative circumstances and individual biological vulnerability. Identifying key risk factors will enable individuals at particularly high risk to be identified, so that strategies can be employed to minimize the risk; and both risk and protective factors will give important insights into the mechanisms of POCD which will enable the development of more effective treatments and better prevention.

By addressing these key questions, the current proposal will clarify the full importance of POCD in people over the age of 70, determine the incidence of subsequent MCI and dementia and examine the impact of a an intra-operative intervention to prevent post operative cognitive dysfunction.

**7. Hypotheses**

a)In an RCT, closer anaesthetic monitoring and anaesthetic management of patients during surgery will improve post-operative cognitive outcomes compared with patients receiving usual anaesthetic monitoring at 7 days and at 3 months post-operatively.

.

b)Twelve months post-operatively, overall cognitive performance and function on every day activities will be significantly more impaired in post-operative patients than in age matched controls.

c) Twelve months post-operatively the incidence of MCI and dementia will be significantly higher in post-operative patients than in age matched controls.

d) A fall in cerebral oxygenation >15% will be associated with a significant increase in the risk of POCD and strategies to correct or minimise this fall with reduce the incidence of POCD

e) Minimisation of cumulative deep anaesthetic time will reduce the incidence of POCD

f) Genes predisposing to dementia, attentional impairments, delirium and abnormal NOS activity will increase the risk of POCD.

**8** **Methods** **Participants, Inclusion and exclusion criteria**:

*Randomized Controlled Trial (RCT)*

One hundred patients, 60 years of age and older, with an American Society of Anesthesiologists (ASA) classification of fitness of 3 or less, undergoing major elective abdominal or orthopaedic surgery under general anaesthesia at King’s College Hospital will participate in the study.

Inclusion criteria

(1)Patients over 60 years of age classified as ASA (American Society of Anesthesiologists) of three or less who were due to undergo major elective abdominal or orthopaedic surgery (2) Mini Mental State Examination (MMSE) score equal to or greater than 23. (3) Adequate English was a pre-requisite to enable consent and to undertake study assessments.

Exclusion criteria

(1) Unable to complete the outcome measures (2) A diagnosis of Alzheimer’s disease or other dementia (3) Individuals undergoing surgical procedures under regional anaesthetics.

Assessments

1/ Electrocardiography (ECG) and laboratory tests as clinically indicated.

2/ Cognitive evaluation: Global cognitive function (Cambridge Assessment for Mental Disorder in the Elderly, section B (CAMCOG-B - , Mini-Mental State Examination -MMSE), memory (CAMCOG memory subscale), executive function (Trail – Making task part A and B) and vigilance accuracy and vigilance reaction time from the Cognitive Drug Research battery as indices of attentional performance and cognitive processing speed pre-operatively, 1 week post surgery and 12 weeks post surgery.

Definitions of POCD and other criteria for cognitive dysfunction will be validated based upon an initial cohort study (appendix 1).

Randomisation

Participants will be randomized according to randomisation lists, stratified by age group (65 to 70, 70 to 75 and over 75) , which will be generated by the study statistician in the statistical program package R. Based upon the list, sealed envelopes containing the randomization codes will be delivered to the operating theatres, and an envelope selected randomly by the anaesthetist. The randomisation envelope will be opened only after the participant’s eligibility and willingness to participate are re-confirmed prior to surgery.

Blinding

The trial will be double blind with the patients, informants and researchers blind to treatment allocation.

Monitoring and Intervention

A bispectral index (BIS) of the electroencephalogram will be used to monitor anaesthetic depth. The target BIS range will be 40-60 (±5).

During and until extubation after the procedure, rSO2 will be continuously monitored using SICO to enable interventions if regional cerebral saturations drop by more than 15% from baseline or to less than 50% intraoperatively, using the following algorithm

1. BP maintained within 10% of baseline value by giving fluids or inotropes to bring the value back to within these limits
2. SpO2 maintained above 95% by increasing the FIO2 to 50%
3. Maintain EtCO2 between 4·5 to 5·5%. avoiding excessive hypercarbia as well as hypocarbia.
4. If there is ongoing moderate to severe haemorrhage consider transfusion if the Hb level is less than 9g.dl-1

If all the above fail to correct a decline then increase the etCO2 to 6% and give 100% O2.

Intervention

In the intervention group the anaesthetist will have full access to the BIS and SICO data to optimize the depth of anesthesia and to identify and facilitate intervention to rectify cerebral oxygen desaturation. In the control group the BIS and SICO data will be collected, but the anaesthetist will be blind to the monitoring data.

Statistical methods

The study is powered as a pilot study. A cohort of 73 patients gives 80% power to the 5% level of significance to detect a between group difference of 0·33 SD. The goal is to enroll 100 participants to allow for drop-outs.

The primary efficacy population will be defined as all patients who were randomised to receive the intra-operative anesthetic intervention or treatment as usual and completed at least one follow-up assessment. Statistical analyses will be undertaken using the SPSS software, version 17.0 (SPSS, Chicago, IL, USA). Two-sided p values of less than 0·05 will be deemed to be statistically significant. A full statistical evaluation protocol will be developed by the study statistician.

Additional Objectives

Fifty age matched controls will be recruited in parallel, drawn from the spouses of people undergoing surgery. The pre-operative assessment will include a standardized evaluation to identify pre-existing delirium (Confusion Assessment Method CAM^29^) or significant cognitive impairment including MMSE <25^30^, and/or the presence of dementia (DSMIIIR criteria, American Psychiatric Association 1987). This will allow additional comparisons between post-operative patients and age matched controls to address hypotheses b-f.

As a biomarker of brain injury,^26^ S100B protein will measured in plasma. Blood samples will be taken from participants at baseline and one day post-surgery. Blood will be collected into K2-EDTA vacutainers and centrifuged at 2,000g for ten minutes. Plasma will be collected and frozen until analysis. S100B concentrations will be measured using a commercially available ELISA (Alpco Diagnostics , Salem, NH, USA) according to the manufacturer’s instructions. A full biomarker analysis protocol will be developed as part of the study.

**9.** **The value of the research to Public health and patient care**: It is clear that Post-Operative Cognitive Decline (POCD) is frequent after major surgical procedures, especially in the oldest age groups. Surprisingly, despite the high incidence rates, awareness is low amongst the medical community and amongst surgical patients; and people are rarely informed of the risk of POCD as part of the consent procedure. Determining the full significance of POCD with respect to longer term cognitive outcomes such as Mild Cognitive Impairment and dementia is essential to enable clear communication of the likely risks and prognosis. Even more importantly elucidating the key contributing factors and the underlying mechanisms is essential so that effective prevention strategies can be assessed.

**10**. **References**:

Beecham and Knapp (2001). in Thornicroft (ed.)Measuring Mental Health Needs, Gaskell, London, 2^nd^ ed, B

**O'Donnell (1983) et al. J. of Clin. Psych. 39:746-8,

**Simpson (1989) Br. J. of Clin Pharm 27: 647-648,

The EuroQol Group (1990) Health Policy 16, 199-208,

**Wimmer-Greinecker et al. (1998) Thorac Cardiovasc Surg 46:207-12,

1. Moller JT, Cluitmans P, Rasmussen LS, Houx P, Rasmussen H, Canet J, Rabbitt P, Jolles J, Larsen K, Hanning CD, Langeron O, Johnson T, Lauven PM, Kristensen PA, Biedler A, van Beem H, Fraidakis O, Silverstein JH, Beneken JE, Gravenstein JS: Long-term postoperative cognitive dysfunction in the elderly ISPOCD1 study. ISPOCD investigators. International Study of Post-Operative Cognitive Dysfunction [see comments]. Lancet 1998; 351: 857-61 Issn: 0140-6736

2. Selwood A, Orrell M: Long term cognitive dysfunction in older people after non-cardiac surgery. Bmj 2004; 328: 120-1

3. Mackensen GB, Gelb AW: Postoperative cognitive deficits: more questions than answers. Eur J Anaesthesiol 2004; 21: 85-8

4. Samra SK, Dy EA, Welch K, Dorje P, Zelenock GB, Stanley JC: Evaluation of a cerebral oximeter as a monitor of cerebral ischemia during carotid endarterectomy. Anesthesiology 2000; 93: 964-70

5. Edmonds HL, Jr.: Detection and treatment of cerebral hypoxia key to avoiding intraoperative brain injuries. J Clin Monit Comput 2000; 16: 69-74

6. Edmonds HL, Jr.: Multi-modality neurophysiologic monitoring for cardiac surgery. Heart Surg Forum 2002; 5: 225-8

7. Edmonds HL, Jr., Ganzel BL, Austin EH, 3rd: Cerebral oximetry for cardiac and vascular surgery. Semin Cardiothorac Vasc Anesth 2004; 8: 147-66

8. Lassnigg A, Hiesmayr M, Keznickl P, Mullner T, Ehrlich M, Grubhofer G: Cerebral oxygenation during cardiopulmonary bypass measured by near-infrared spectroscopy: effects of hemodilution, temperature, and flow. J Cardiothorac Vasc Anesth 1999; 13: 544-8

9. Plachky J, Hofer S, Volkmann M, Martin E, Bardenheuer HJ, Weigand MA: Regional cerebral oxygen saturation is a sensitive marker of cerebral hypoperfusion during orthotopic liver transplantation. Anesth Analg 2004; 99: 344-9, table of contents

10. Luer MS, Dujovny M, Slavin KV, Hernandez-Avila G, Ausman JI: Regional cerebral oxygen saturation during intra-arterial papaverine therapy for vasospasm: case report. Neurosurgery 1995; 36: 1033-6

11. Casati A, Fanelli G, Pietropaoli P, Proietti R, Tufano R, Danelli G, Fierro G, De Cosmo G, Servillo G: Continuous monitoring of cerebral oxygen saturation in elderly patients undergoing major abdominal surgery minimizes brain exposure to potential hypoxia. Anesth Analg 2005; 101: 740-7, table of contents

12. Beals JK, Carter LB, Jevtovic-Todorovic V: Neurotoxicity of nitrous oxide and ketamine is more severe in aged than in young rat brain. Ann N Y Acad Sci 2003; 993: 115; discussion 123-4

13. Olney JW: New insights and new issues in developmental neurotoxicology. Neurotoxicology 2002; 23: 659-68

14. Buhre W, Rossaint R: Perioperative management and monitoring in anaesthesia. Lancet 2003; 362: 1839-46

15. Monk TG, Saini V, Weldon BC, Sigl JC: Anesthetic management and one-year mortality after noncardiac surgery. Anesth Analg 2005; 100: 4-10

16. Albertin A, Casati A, Bergonzi P, Fano G, Torri G: Effects of two target-controlled concentrations (1 and 3 ng/ml) of remifentanil on MAC(BAR) of sevoflurane. Anesthesiology 2004; 100: 255-9

17. Yoshitani K, Kawaguchi M, Iwata M, Sasaoka N, Inoue S, Kurumatani N, Furuya H: Comparison of changes in jugular venous bulb oxygen saturation and cerebral oxygen saturation during variations of haemoglobin concentration under propofol and sevoflurane anaesthesia. Br J Anaesth 2005; 94: 341-6

18. Linstedt U, Meyer O, Kropp P, Berkau A, Tapp E, Zenz M: Serum concentration of S-100 protein in assessment of cognitive dysfunction after general anesthesia in different types of surgery. Acta Anaesthesiol Scand 2002; 46: 384-9

19. Iohom G, Szarvas S, Larney V, O'Brien J, Buckley E, Butler M, Shorten G: Perioperative plasma concentrations of stable nitric oxide products are predictive of cognitive dysfunction after laparoscopic cholecystectomy. Anesth Analg 2004; 99: 1245-52, table of contents

20. de Vries TJ, Smeets M, de Graaf R, Hou-Jensen K, Brocker EB, Renard N, Eggermont AM, van Muijen GN, Ruiter DJ: Expression of gp100, MART-1, tyrosinase, and S100 in paraffin-embedded primary melanomas and locoregional, lymph node, and visceral metastases: implications for diagnosis and immunotherapy. A study conducted by the EORTC Melanoma Cooperative Group. J Pathol 2001; 193: 13-20

21. Foerch C, du Mesnil de Rochemont R, Singer O, Neumann-Haefelin T, Buchkremer M, Zanella FE, Steinmetz H, Sitzer M: S100B as a surrogate marker for successful clot lysis in hyperacute middle cerebral artery occlusion. J Neurol Neurosurg Psychiatry 2003; 74: 322-5

22. Ashraf S, Bhattacharya K, Tian Y, Watterson K: Cytokine and S100B levels in paediatric patients undergoing corrective cardiac surgery with or without total circulatory arrest. Eur J Cardiothorac Surg 1999; 16: 32-7

23. Rothermundt M, Peters M, Prehn JH, Arolt V: S100B in brain damage and neurodegeneration. Microsc Res Tech 2003; 60: 614-32

24. Elimian A, Figueroa R, Verma U, Visintainer P, Sehgal PB, Tejani N: Amniotic fluid neuron-specific enolase: a role in predicting neonatal neurologic injury? Obstet Gynecol 1998; 92: 546-50

25. Lizasoain I, Moro MA, Knowles RG, Darley-Usmar V, Moncada S: Nitric oxide and peroxynitrite exert distinct effects on mitochondrial respiration which are differentially blocked by glutathione or glucose. Biochem J 1996; 314 ( Pt 3): 877-80

26. Satoi S, Kamiyama Y, Kitade H, Kwon AH, Yoshida H, Nakamura N, Takai S, Uetsuji S, Okuda K, Hara K, Takahashi H: Prolonged decreases in plasma nitrate levels at early postoperative phase after hepato-pancreato-biliary surgery. J Lab Clin Med 1998; 131: 236-42

27. Brett SJ, Quinlan GJ, Mitchell J, Pepper JR, Evans TW: Production of nitric oxide during surgery involving cardiopulmonary bypass. Crit Care Med 1998; 26: 272-8

28. Khaldi A, Chiueh CC, Bullock MR, Woodward JJ: The significance of nitric oxide production in the brain after injury. Ann N Y Acad Sci 2002; 962: 53-9

29. Inouye SK, van Dyck CH, Alessi CA, Balkin S, Siegal AP, Horwitz RI: Clarifying confusion: the confusion assessment method. A new method for detection of delirium. Ann Intern Med 1990; 113: 941-8

30. Folstein MF, Folstein SE, McHugh PR: "Mini-mental state". A practical method for grading the cognitive state of patients for the clinician. J Psychiatr Res 1975; 12: 189-98

31. Roth M, Tym E, Mountjoy CQ, Huppert FA, Hendrie H, Verma S, Goddard R: CAMDEX. A standardised instrument for the diagnosis of mental disorder in the elderly with special reference to the early detection of dementia. Br J Psychiatry 1986; 149: 698-709

32. O'Donnell WE, Reynolds DM, De Soto CB: Neuropsychological impairment scale (NIS): initial validation study using trailmaking test (A & B) and WAIS digit symbol (scaled score) in a mixed grouping of psychiatric, neurological, and normal patients. J Clin Psychol 1983; 39: 746-8

33. Golden: Stroop color and word test. A manual for clinical and experimental uses. Chicago, Stoelting, 1978

34. Burton EJ, Kenny RA, O'Brien J, Stephens S, Bradbury M, Rowan E, Kalaria R, Firbank M, Wesnes K, Ballard C: White matter hyperintensities are associated with impairment of memory, attention, and global cognitive performance in older stroke patients. Stroke 2004; 35: 1270-5

35. Ballard CG, Aarsland D, McKeith I, O'Brien J, Gray A, Cormack F, Burn D, Cassidy T, Starfeldt R, Larsen JP, Brown R, Tovee M: Fluctuations in attention: PD dementia vs DLB with parkinsonism. Neurology 2002; 59: 1714-20

36. Petersen RC, Smith GE, Waring SC, Ivnik RJ, Kokmen E, Tangelos EG: Aging, memory, and mild cognitive impairment. Int Psychogeriatr 1997; 9 Suppl 1: 65-9

37. Byrne LM, Wilson PM, Bucks RS, Hughes AO, Wilcock GK: The sensitivity to change over time of the Bristol Activities of Daily Living Scale in Alzheimer's disease. Int J Geriatr Psychiatry 2000; 15: 656-61

38. Alexopoulos GS, Abrams RC, Young RC, Shamoian CA: Cornell Scale for Depression in Dementia. Biol Psychiatry 1988; 23: 271-84

39. Rasmussen LS, Christiansen M, Eliasen K, Sander-Jensen K, Moller JT: Biochemical markers for brain damage after cardiac surgery - time profile and correlation with cognitive dysfunction. Acta Anaesthesiol Scand 2002; 46: 547-551

40. Gao F, Harris DN, Sapsed-Byrne S: Time course of neurone-specific enolase and S-100 protein release during and after coronary artery bypass grafting. Br J Anaesth 1999; 82: 266-7

41. Wernicke C, Samochowiec J, Schmidt LG, Winterer G, Smolka M, Kucharska-Mazur J, Horodnicki J, Gallinat J, Rommelspacher H: Polymorphisms in the N-methyl-D-aspartate receptor 1 and 2B subunits are associated with alcoholism-related traits. Biol Psychiatry 2003; 54: 922-8

42. Sano A, Kondoh K, Kakimoto Y, Kondo I: A 40-nucleotide repeat polymorphism in the human dopamine transporter gene. Hum Genet 1993; 91: 405-6

43. Yen T, Nightingale BN, Burns JC, Sullivan DR, Stewart PM: Butyrylcholinesterase (BCHE) genotyping for post-succinylcholine apnea in an Australian population. Clin Chem 2003; 49: 1297-308

44. Green AJ, Keir G, Thompson EJ: A specific and sensitive ELISA for measuring S-100b in cerebrospinal fluid. J Immunol Methods 1997; 205: 35-41

45. Petzold A, Keir G, Green AJ, Giovannoni G, Thompson EJ: A specific ELISA for measuring neurofilament heavy chain phosphoforms. J Immunol Methods 2003; 278: 179-90

46. Berger RP, Pierce MC, Wisniewski SR, Adelson PD, Clark RS, Ruppel RA, Kochanek PM: Neuron-specific enolase and S100B in cerebrospinal fluid after severe traumatic brain injury in infants and children. Pediatrics 2002; 109: E31

DG 13 5 2006

**Appendix 1**: Data collected for a cohort study on Post Operative delirium (n=100) will be evaluated, to provide pilot data to enable optimization study criteria for POCD and other indices of cognitive dysfunction in the RCT. Age at surgery was a significant predictor of recovery to baseline MMSE (t = 2.3 p = 0.023), with an odds ratio of recovery >2 for the under 80’s. Simple Reaction Time (SRT) showed the lowest recovery rate, with only 45% of patients recovering to their pre-operative level within the follow up period of twelve months. Importantly, slower pre-operative SRT (t=4.1 p=<0.0001) and Choice Reaction Time (CRT) (t=3.7 p=<0.0001 ) were significant predictors of “non-recovery”.

Table 1: Recovery rates for Global Cognition and Attention

|  | Follow Up Visits | | | | |
| --- | --- | --- | --- | --- | --- |
|  | 3 Days | 7 Days | 3 Months | 6 Months | 12 Months |
| MMSE | 45 | 74 | 91 | 92 | 92 |
| SRT | 23 | 36 | 40 | 44 | 45 |
| CRT | 27 | 58 | 68 | 77 | 77 |

Patients recovered to baseline (%).

**C Details of lead investigators**

**1.** **Lead applicant / investigator** (to whom all correspondence will be addressed)

| Surname: Ballard | Title: Professor |
| --- | --- |
| Forename: Clive | Speciality: Old Age Psychiatry |
| Post held: Professor of Age Related Diseases | E-mail: [clive.ballard@kcl.ac.uk](mailto:clive.ballard@kcl.ac.uk) |
| Department: Institute of Psychiatry | |
| Organisation: King’s College London | |
| Official address:  Wolfson Centre for Age Related Diseases  Guy’s Campus  King’s College London | Postcode: SE1 1UL  Tel. No. / Ext.: 0207 8486568  Fax No.: 02078486145 |

**2.** **Other investigators:**

| Surname: Green | Title: Dr. |  |
| --- | --- | --- |
| Forename: David | Speciality: Anaesthetics |  |
| Post held: Consultant Anaesthetist | E-mail: [david.w.green@kcl.ac.uk](mailto:david.w.green@kcl.ac.uk) |  |
| Department: Anaesthetics, Intensive Care and Pain Relief | |  |
| Organisation: King’s College Hospital NHS Trust | |  |
| Official address:  Denmark Hill  London | Postcode: SE5 9RS  Tel. No. / Ext.: 0207346 3154/3919  Fax No.: |  |

| Surname: Wesnes | Title: Professor |  |
| --- | --- | --- |
| Forename: Keith | Speciality: Cognitive Psychopharmacology |  |
| Post held: Visiting Professor | E-mail: [keithw@cdr.org.uk](mailto:keithw@cdr.org.uk) |  |
| Department: Human Cognitive Neuroscience Unit | |  |
| Organisation: , Northumbria University | |  |
| Official address:  CDR Ltd, Gatehampton Road  Goring-on-Thames, | Postcode: RG8 0EN  Tel. No. / Ext.: 01491 878700  Fax No.: |  |

| Surname: Jones | Title: |  |
| --- | --- | --- |
| Forename: Emma | Speciality: |  |
| Post held: Research Associate | E-mail: |  |
| Department:Wolfson Centre for Age Related Disease | |  |
| Organisation: King’s College London | |  |
| Official address: Wolfson CARD,  King’s College London,  Guy’s Campus,  London | Postcode: SE1 1UL  Tel. No. / Ext.: 0207 848 6156  Fax No.: 0207 848 6145 |  |
